# Supplementary material for: Flow-mediated modulation of the endothelial cell lipidome
Source: Front Physiol. 2024 Jul 24;15:1431847. doi: 10.3389/fphys.2024.1431847 (PMC11307263; doi:10.3389/fphys.2024.1431847)
Supplement: Supplementary file 1 [file Presentation1.pdf]

Supplementary Figures

Flow-Mediated Modulation of the Endothelial Cell Lipidome

Authors: Soon-Gook Hong, John P. Kennelly, Kevin J. Williams, Steven J. Bensinger and Julia J. Mack*

* Correspondence:
Julia J. Mack
Department of Medicine, Division of Cardiology
University of California, Los Angeles
650 Charles E. Young Drive South, Los Angeles, 90095, USA.
Tel: (310) 825-5749
Email: jmack@mednet.ucla.edu

This file contains the following:
Supplementary Figures S1 − S10

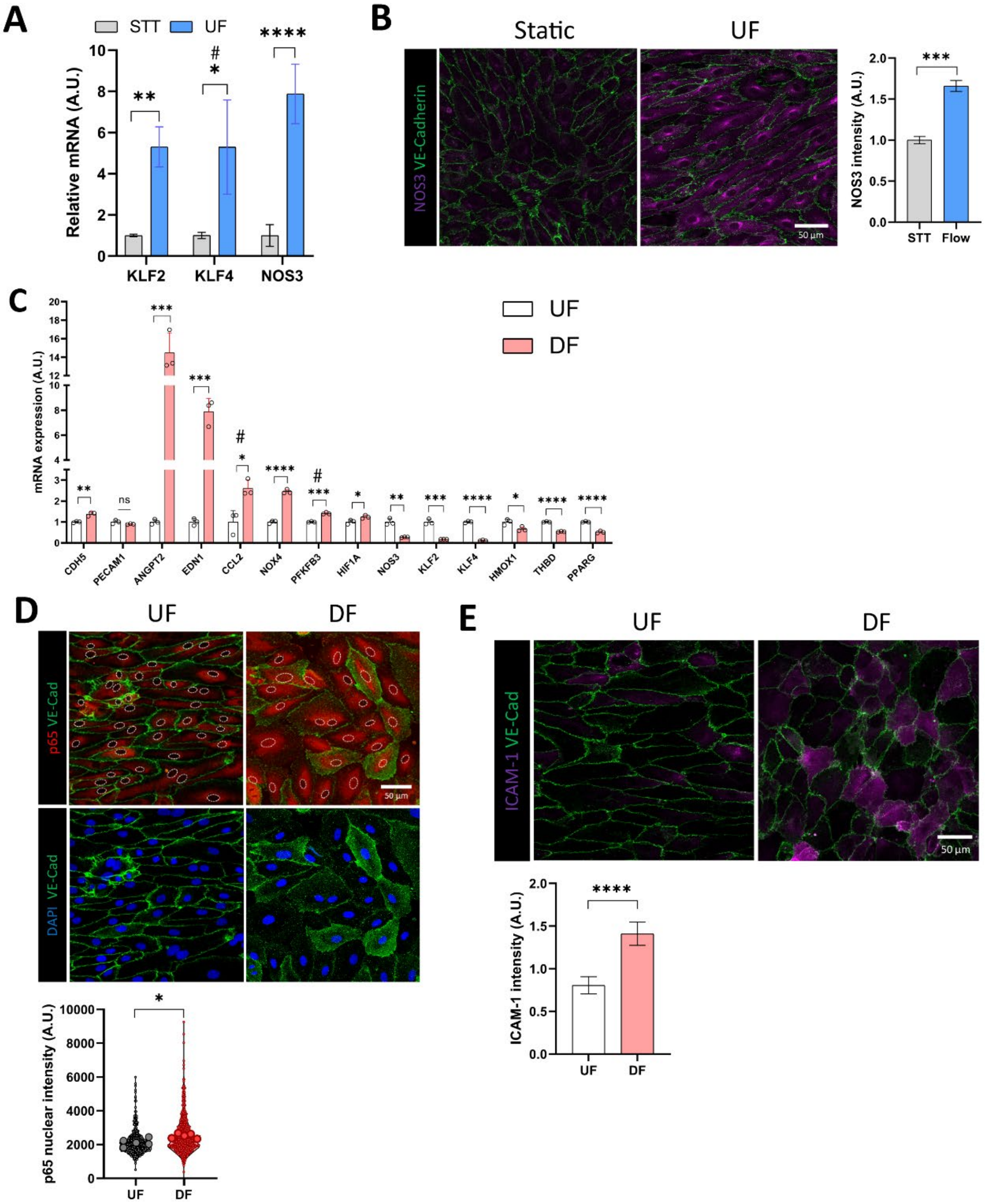

Supplementary Figure S1: Endothelial phenotype is altered for UF versus DF

(A) Expression levels of flow-responsive genes for HAECs exposed to UF or static conditions for 48 h (n=3). **(B)** Confocal imaging of total eNOS expression in HAECs under static or UF. Elevation of eNOS protein was measured by immunostaining and quantification of fluorescence intensity (n=3). Green = VE-Cadherin, Magenta = eNOS. **(C)** Expression levels of genes known to change in response to DF vs. UF (n=3). **(D)** NF-κB p65 nuclear localization, an indicator of NF-κB-mediated inflammation, was enhanced for HAECs under DF. Red = p65, Green = VE-Cadherin. **(E)** Representative confocal images of ICAM-1 and VE-Cadherin immunostaining of HAECs exposed to UF vs. DF. ICAM-1 protein expression was greater under DF as quantified by mean fluorescence intensity (n=5). Bar graph shown as mean ± SD. *p<0.05, **p<0.01, ***p<0.001, ****p<0.001, ns=not significant by two-tailed unpaired *t*-test. #p<0.05 by one-tailed Mann-Whitney U test.

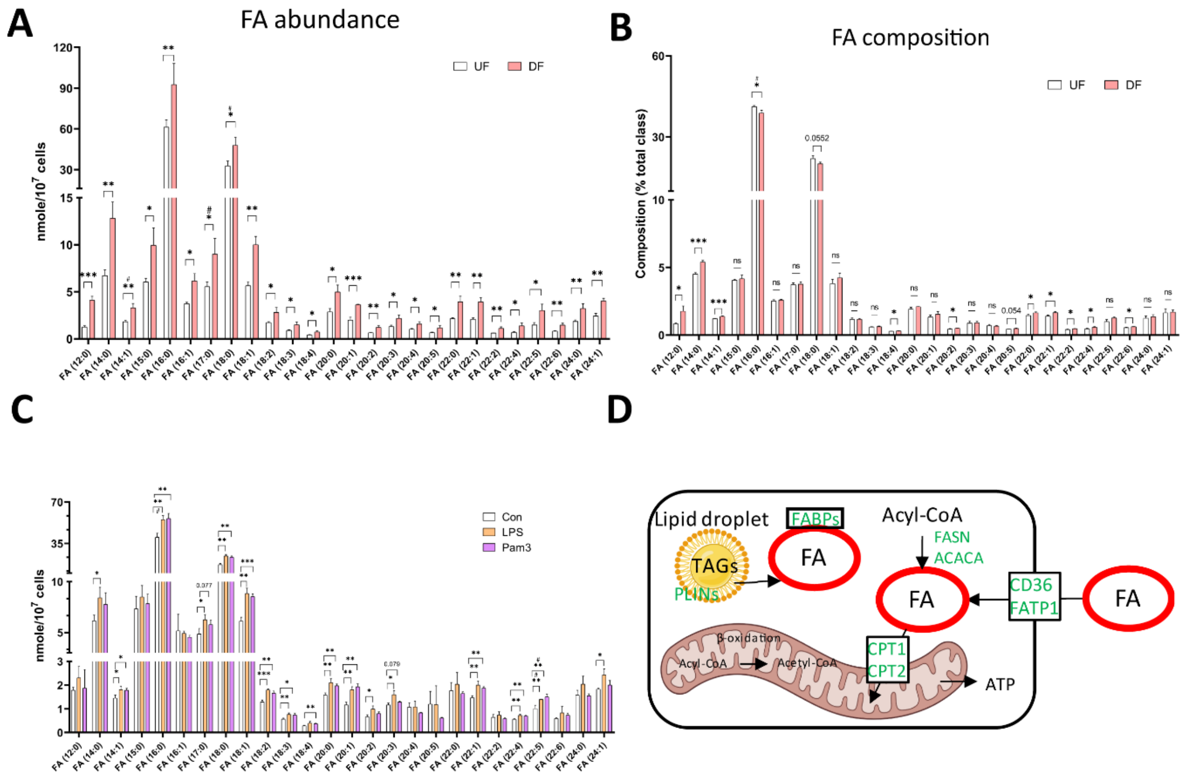

Supplementary Figure S2: Fatty acid abundance and composition in HAECs under DF versus UF

(**A**) Bar plot of fatty acid abundance in HAECs exposed to UF vs. DF (n=3). **(B)** Bar plot of fatty acid composition in HAECs under UF vs. DF (n=3). **(C)** Bar graph of fatty acid abundance in HAECs exposed to vehicle, LPS, and Pam3. Bar graph shown as mean ± SD (n=3). **(D)** Schematic diagram of proteins that regulate fatty acid abundance in cells. Fatty acid abundance is regulated by proteins that modulate the uptake, synthesis, and transport of fatty acids, as well as proteins that hydrolyze fatty acids from neutral lipids and phospholipids. *p<0.05, **p<0.01, ***p<0.001, ****p<0.001, ns=not significant by two-tailed unpaired *t*-test. #p<0.05 by one-tailed Mann-Whitney U test.

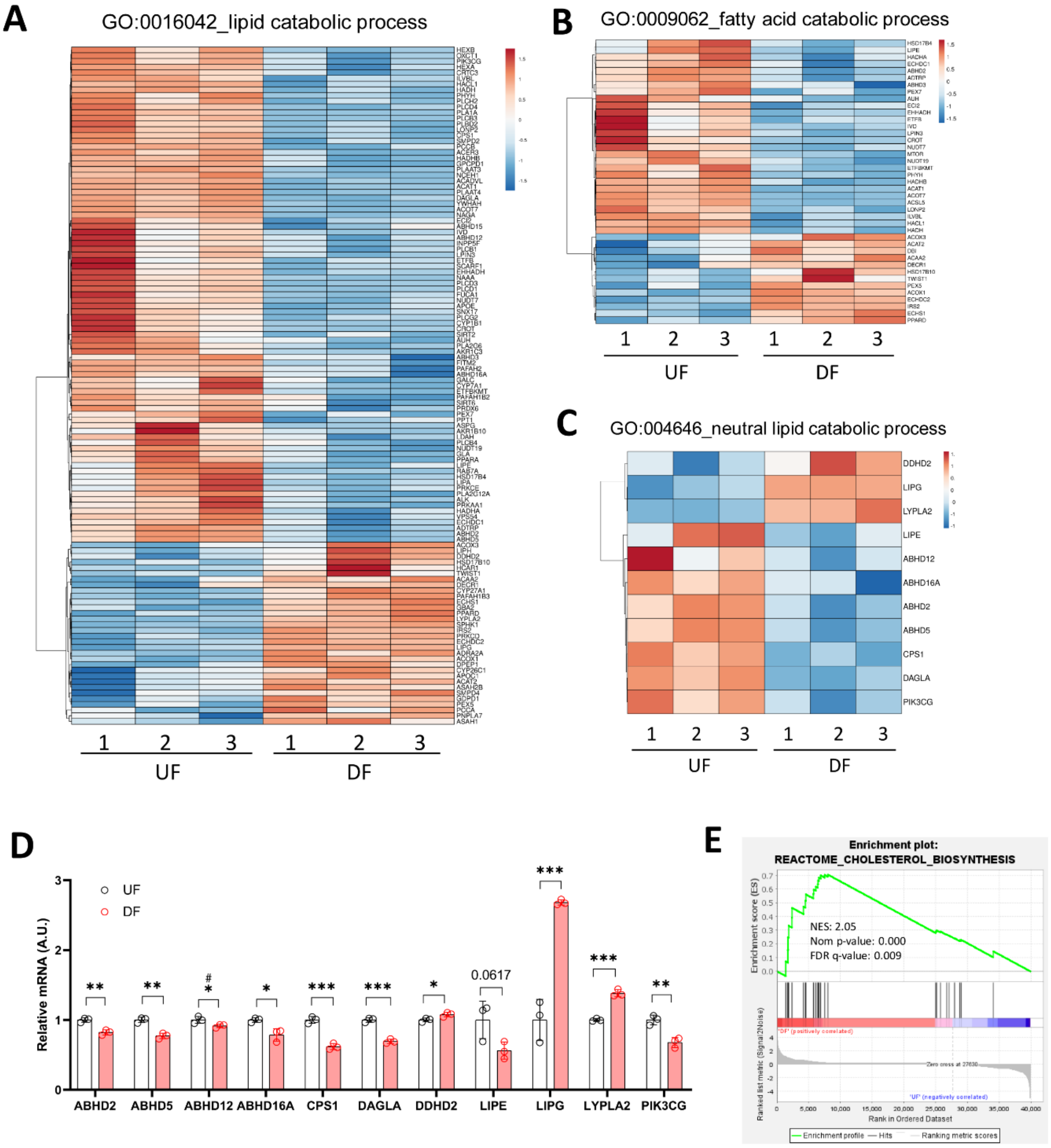

Supplementary Figure S3: Expression of genes involved in lipid catabolic process

(**A**) Heatmap of genes involved in lipid catabolic process from gene ontology (GO) database (GO:0016042). (**B**) Heatmap of genes involved in fatty acid catabolic process from gene ontology (GO) database (GO:0009062). (**C**) Heatmap of genes involved in neutral lipid catabolic process from gene ontology (GO) database (GO:004646). **(D)** Bar plot using the values from (B) (n=3). (**E**) GSEA enrichment plot using REACTOME database. Genes involved in cholesterol biosynthesis pathway were significantly enriched under DF vs. UF. NES = normalized enrichment score. norm p-value = Normalized p-value. FDR q-value = false discovery rate q-value. Bar graph shown as mean ± SD. *p<0.05, **p<0.01, ***p<0.001, ns=not significant by two-tailed unpaired *t*-test. #p<0.05 by one-tailed Mann-Whitney U test.

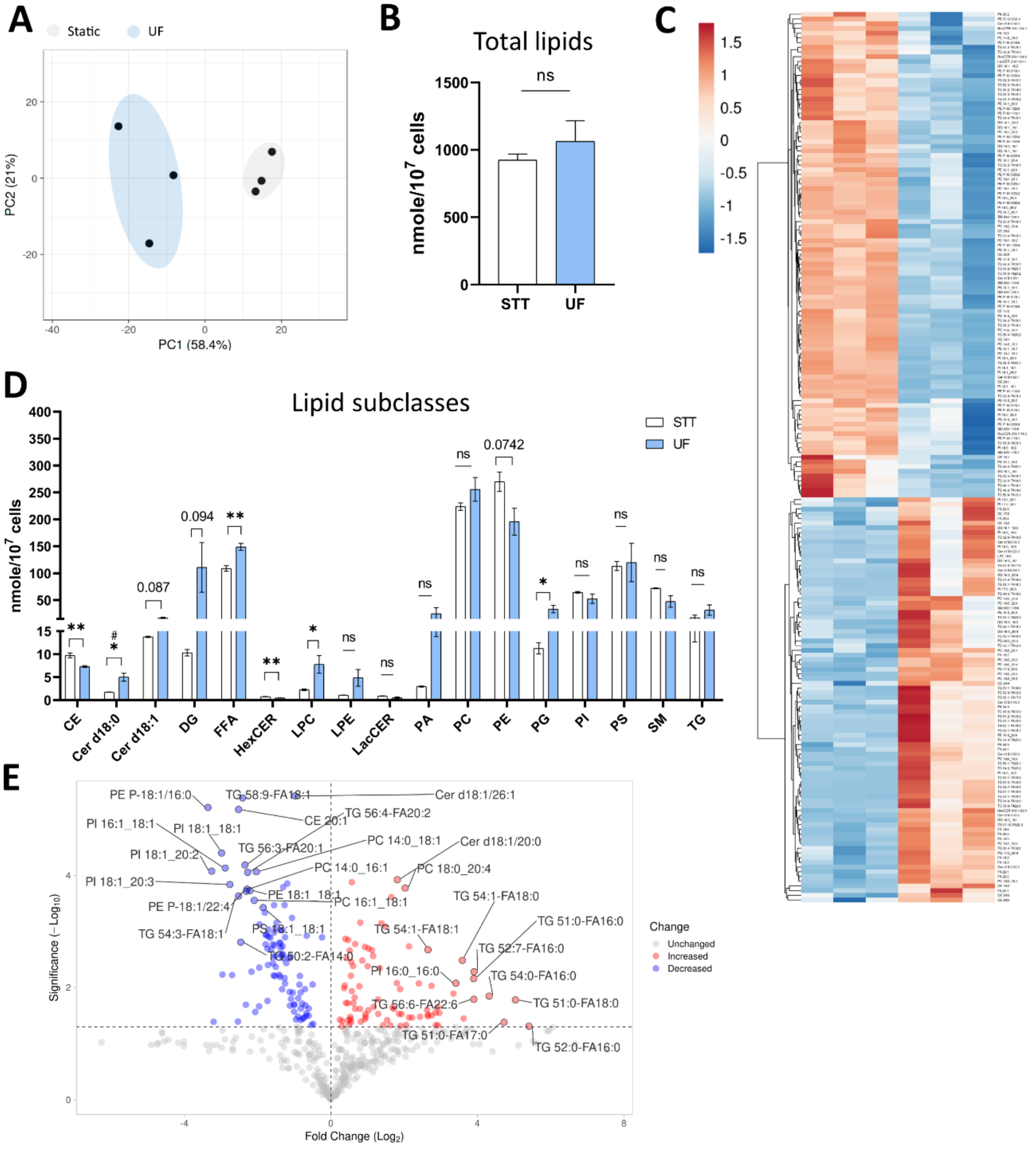

Supplementary Figure S4: Changes in lipid abundance for HAECs exposed to static versus UF conditions

(**A**) PCA plot of lipid species comparison for static and UF. Grey = Statically cultured HAECs, Blue = UF-exposed HAECs. **(B)** Bar graph for total lipid abundance (n=3). **(C)** Heatmap of altered lipid species under UF vs. static cultured HAECs. **(D)** Bar plot of lipid subclass abundance under static and UF (n=3). **(E)** Volcano plot of lipid species for HAECs exposed to UF vs. static conditions. Bar graph shown as mean ± SD. *p<0.05, **p<0.01, ns=not significant by two-tailed unpaired *t*-test. #p<0.05 by one-tailed Mann-Whitney U test.

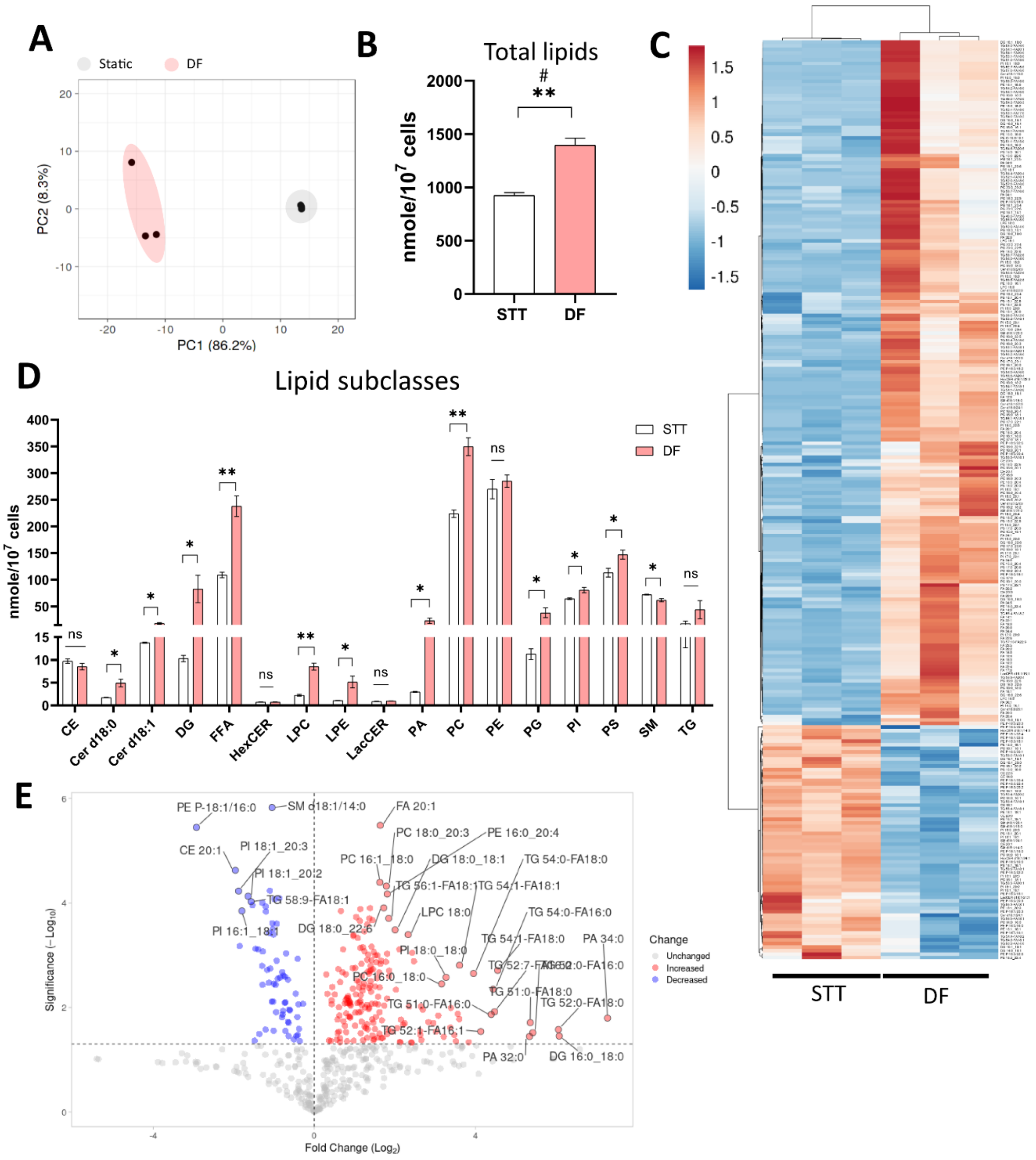

Supplementary Figure S5: Lipid abundance for HAECs exposed to static versus DF conditions

(**A**) PCA plot using lipid species. Grey = Statically cultured HAECs, Red = DF-exposed HAECs. **(B)** Bar graph for total lipid abundance (n=3). **(C)** Heatmap of altered lipid species for HAECs under DF vs. static conditions. (n=3) **(D)** Bar plot of lipid subclass abundance in HAECs cultured under static vs. DF (n=3). **(E)** Volcano plot of lipid species for HAECs under DF vs. static. Bar graph shown as mean ± SD. *$p<0.05$, **$p<0.01$, ns=not significant by two-tailed unpaired t-test. #$p<0.05$ by one-tailed Mann-Whitney U test.

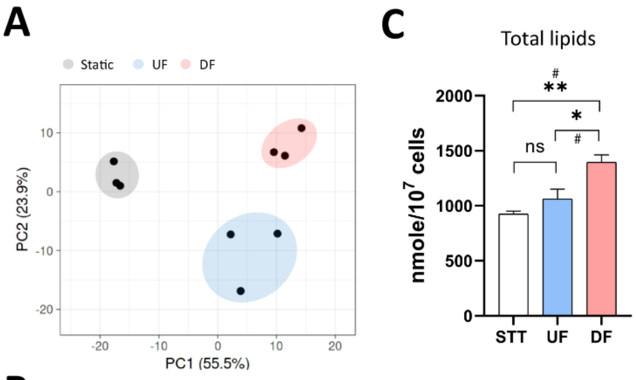

B

Top 20 PCA loadings for **PC1**		Top 20 PCA loadings for **PC2**	
1	PC 18:0_20:3	1	PE O-18:0/18:1
2	Cer d18:1/20:0	2	PS 16:1_18:0
3	Cer d18:1/22:0	3	PI 16:0_18:1
4	PC 18:0_18:2	4	SM d18:1/18:1
5	PE P-18:1/16:0	5	PE P-18:0/18:1
6	CE 20:1	6	TG 56:4-FA20:1
7	TG 54:0-FA16:0	7	TG 51:2-FA16:0
8	PC 16:1_18:0	8	TG 50:2-FA14:1
9	PE 18:0_22:6	9	PS 18:0_18:1
10	Cer d18:1/18:0	10	PE P-18:0/16:1
11	DG 18:0_18:1	11	PE P-18:0/20:3
12	PE 18:0_20:4	12	TG 56:3-FA18:1
13	TG 54:1-FA18:0	13	PE P-18:1/18:1
14	TG 52:7-FA16:0	14	TG 53:2-FA18:1
15	TG 51:0-FA16:0	15	TG 56:3-FA20:2
16	CE 20:3	16	TG 54:2-FA20:1
17	PI 18:1_20:3	17	PI 18:0_20:2
18	TG 54:1-FA18:1	18	PC 18:2_20:4
19	FA 18:0	19	PE P-16:0/18:1
20	PI 16:0_16:0	20	PC 15:0_16:1

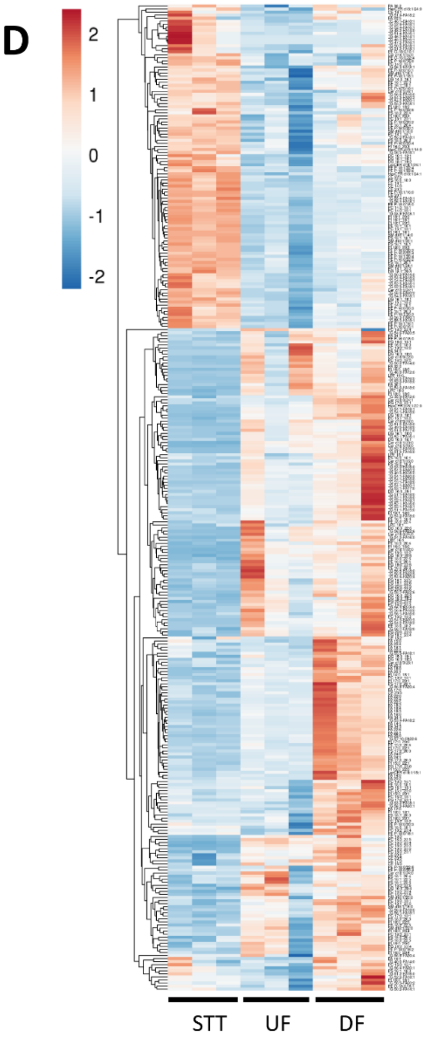

Supplementary Figure S6: Lipid abundance for HAECs exposed to flow (UF & DF) versus static conditions

(**A**) PCA plot using lipid species. Grey = Statically cultured HAECs, Blue = UF-exposed HAECs, Red = DF-exposed HAECs. **(B)** Top 20 PCA loadings for PC1 and PC2. **(C)** Bar graph for total lipid abundance (n=3). **(D)** Heatmap of altered lipid species for HAECs cultured under static vs. UF vs. DF conditions. Bar graph shown as mean ± SD. *p<0.05, **p<0.01, ns=not significant by one-way ANOVA. #p<0.05 by one-tailed Mann-Whitney U test.

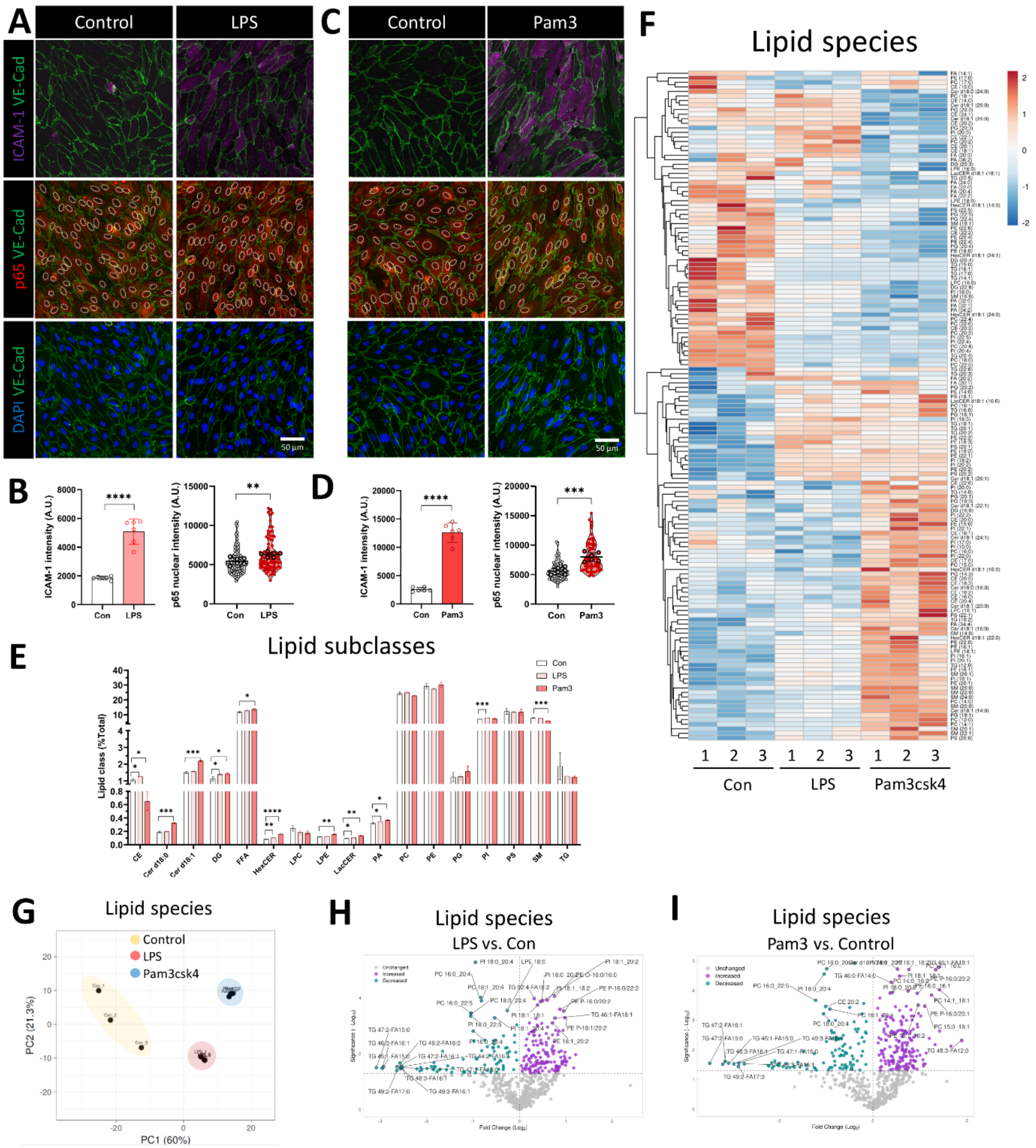

Supplementary Figure S7: Differential lipid composition in response to inflammatory agonist exposure

(A) Confocal imaging of ICAM-1 expression and NF-κB p65 nuclear localization in HAECs in response to control vs. LPS treatment (n=6). Elevation of ICAM-1 expression and p65 nuclear localization were shown in HAECs stimulated with LPS vs. control. Green = VE-Cadherin, Magenta = ICAM-1, Red = p65. **(B)** Dot plots of ICAM-1 and p65 nuclear intensity in control vs. LPS-treated HAECs (n=6). **(C)** Confocal imaging of ICAM-1 and NF-κB p65 nuclear localization in HAECs in response to control vs. Pam3 treatment (n=6). Increased ICAM-1 protein expression and p65 nuclear localization were shown in HAECs stimulated with Pam3 vs. control. Green = VE-Cadherin, Magenta = ICAM-1, Red = p65. **(D)** Dot plots of ICAM-1 and p65 nuclear intensity in control vs. Pam3-treated HAECs (n=6). (**E**) Bar plot of lipid subclass composition (subclass / total lipid) (n=3). **(F)** Heatmap of altered lipid species composition in HAECs exposed to vehicle (control), LPS, and Pam3. **(G)** PCA plot using lipid species composition. **(H)** Volcano plot of lipid species altered in composition under LPS vs. control. **(I)** Volcano plot of lipid species changed in composition under Pam3 vs. control. Bar graph shown as mean ± SD. *p<0.05, **p<0.01, ***p<0.001, ****p<0.001, ns=not significant by two-tailed unpaired *t*-test.

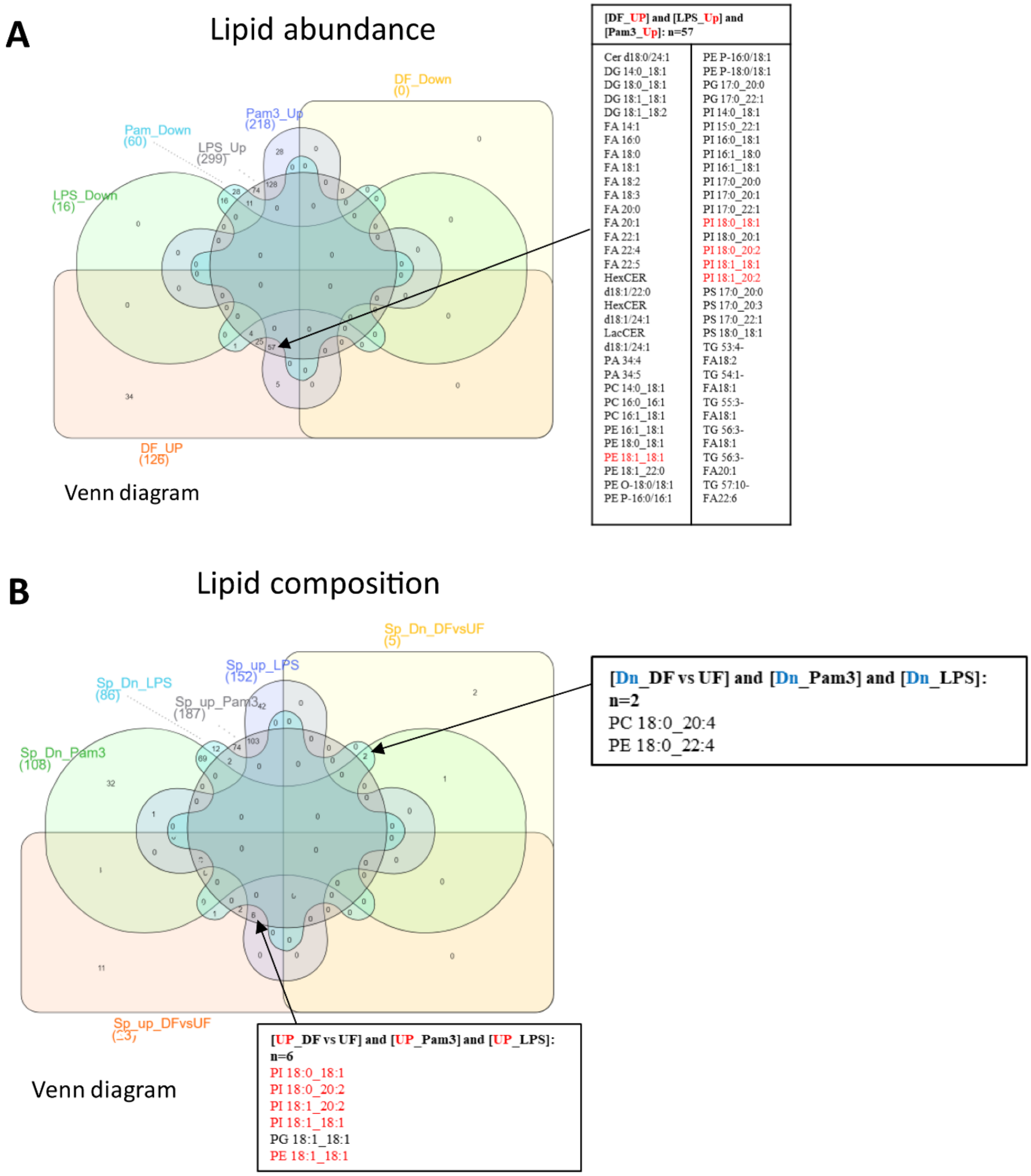

Supplementary Figure S8: Specific lipid species are changed in response to DF and inflammatory agonist exposure

(**A**) Cross analysis using lipid abundance via InteractiVenn. (**B**) Cross analysis using lipid composition via InteractiVenn.

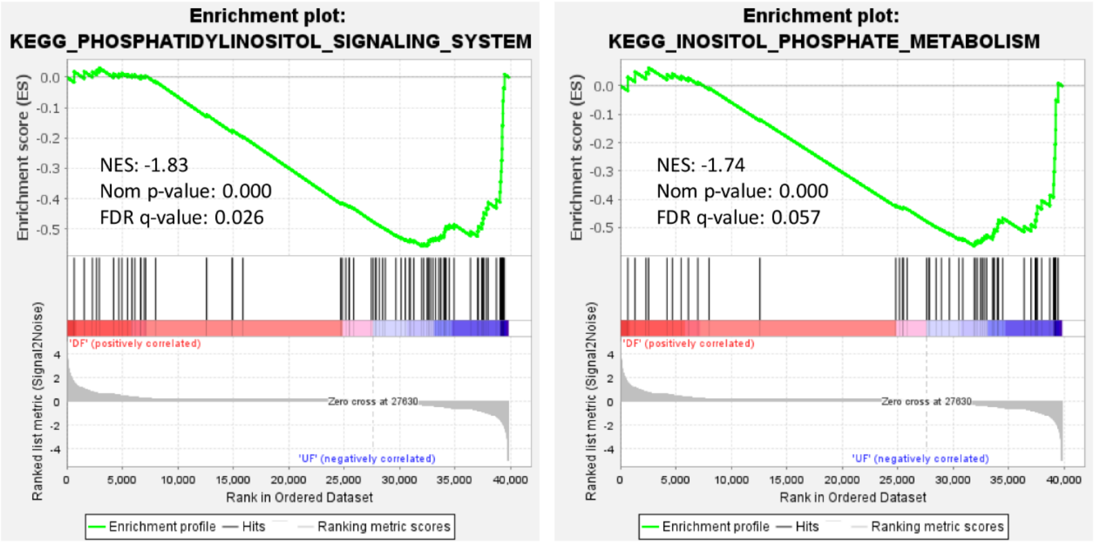

Supplementary Figure S9: Phosphatidylinositol signaling and metabolism pathways are altered under DF

GSEA enrichment plots using KEGG database. Genes involved in phosphatidylinositol (PI) signaling and inositol phosphate metabolism were reduced for HAECs under DF compared to HAECs under UF. NES = normalized enrichment score. norm p-value = Normalized p-value. FDR q-value = false discovery rate q-value.

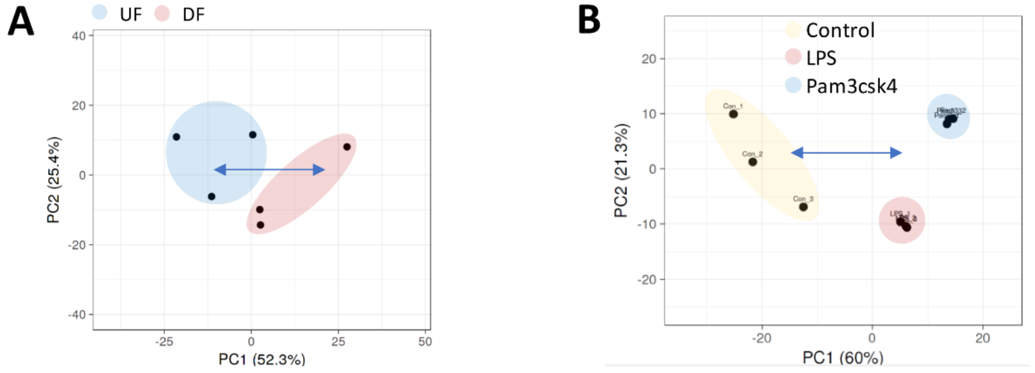

C Top 20 phospholipids in PCA loadings for PC1 (UF vs DF)

Rank	PCA loadings	Rank	PCA loadings
1	PE 16:0_**18:1**	11	PS 18:0_18:2
2	PS 16:0_**18:1**	12	PE O-18:0/**16:1**
3	PC 16:0_**16:1**	13	PE O-16:0/**18:1**
4	PC 16:1_17:0	14	PI 16:0_**18:1**
5	PG 18:1_20:0	15	PG 16:1_**18:1**
6	PE 16:1_18:0	16	PC 16:1_18:2
7	PE 16:0_**16:1**	17	PC 14:0_**16:1**
8	PI 16:1_18:0	18	PE 18:0_**18:1**
9	PE P-18:1/**16:1**	19	PC 15:0_**16:1**
10	PS 16:0_**16:1**	20	PI 18:0_18:2

D Incorporated FA at the sn-2 position in Top 20 phospholipids in PCA loadings for PC1 (UF vs DF)

C no.	Fatty acid	Count
16:1	Palmitoleic Acid	7
17:0	Heptadecanoic acid	1
18:0	Stearic acid	2
18:1	Oleic acid	6
18:2	Linoleic acid	3
20:0	Arachidic acid	1

FA at sn-2	#	%
Saturated	4	20
**Monounsaturated**	**13**	**65**
Polyunsaturated	3	15
Total	20	

F Top 20 phospholipids in PCA loadings for PC1 (Con vs LPS vs Pam3)

Rank	PCA loadings	Rank	PCA loadings
1	PE P-18:1/**20:1**	11	PE P-18:0/**20:1**
2	PS 17:0_20:0	12	PI 18:1_**20:1**
3	PS 16:0_**18:1**	13	PE 18:0_18:2
4	PI 18:1_18:2	14	PE 18:1_18:2
5	PE P-16:0/20:2	15	PI 16:1_**18:1**
6	PI 18:1_**18:1**	16	PI 18:0_**18:1**
7	PS 18:1_**18:1**	17	PI 17:0_20:0
8	PI 16:1_18:0	18	PI 18:0_18:2
9	PC 14:0_16:0	19	PG 16:1_**18:1**
10	PE P-16:0/22:2	20	PE P-18:1/16:0

G Incorporated FA at the sn-2 position in Top 20 phospholipids in PCA loadings for PC1 (Con vs LPS vs Pam3)

C no.	Fatty acid	Count
16:0	Palmitic acid	2
18:0	Stearic acid	1
18:1	Oleic acid	6
18:2	Linoleic acid	4
20:0	Arachidic acid	2
20:1	Gadelaidic acid	3
20:2	Eicosadienoic acid	1
22:2	Docosadienoic acid	1

FA at sn-2	#	%
Saturated	5	25
**Monounsaturated**	**9**	**45**
Polyunsaturated	6	30
Total	20	

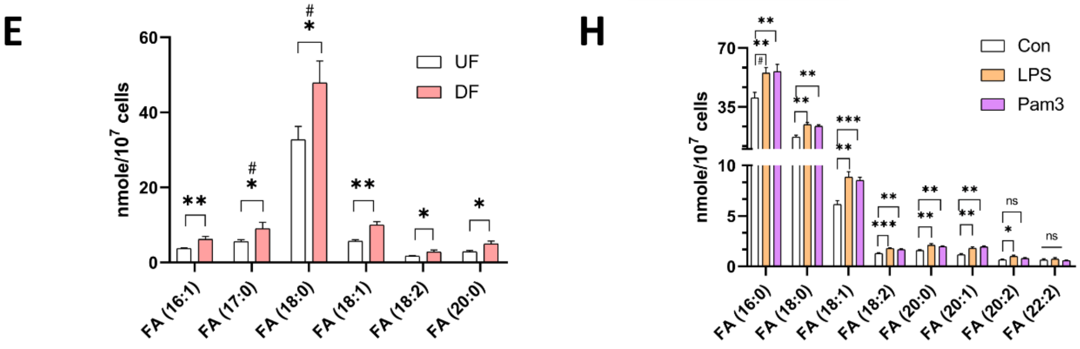

Supplementary Figure S10: Monounsaturated fatty acids were frequently incorporated into phospholipids at the sn-2 position for HAECs exposed to DF and inflammatory agonists

(**A**) PCA plot of lipid species (by abundance) for HAECs exposed to UF vs. DF. **(B)** PCA plot of lipid species (by abundance) for HAECs after vehicle (control), LPS, and Pam3 treatment. **(C)** Table listing the top 20 phospholipids for PC1 of PCA plot in (A). **(D)** Tables listing the incorporated fatty acid at the sn-2 position and percentage of each type of fatty acid in the top 20 phospholipids for PC1 (PCA of UF vs. DF). **(E)** Abundance of fatty acids identified in (D) (n=3). **(F)** Table of the top 20 phospholipids for PC1 (PCA of vehicle vs. LPS vs. Pam3). **(G)** Tables showing the incorporated fatty acid at the sn-2 position and percentage of each type of fatty acid in the top 20 phospholipids for PC1 (Control vs. LPS vs. Pam3). **(H)** Abundance of fatty acids identified in (G) (n=3). Bar graph shown as mean ± SD. *p<0.05, **p<0.01, ***p<0.001, ns=not significant by two-tailed unpaired *t*-test. #p<0.05 by one-tailed Mann-Whitney U test.
